# Late Developing Supernumeraries in a Case of Nonsyndromic Multiple Supernumerary Teeth

**DOI:** 10.1155/2015/840460

**Published:** 2015-01-11

**Authors:** Mine Bozkurt, Tugba Bezgin, Ayşegül Tüzüner Öncül, Rukiye Göçer, Şaziye Sarı

**Affiliations:** ^1^Department of Paediatric Dentistry, Faculty of Dentistry, Ankara University, 06500 Ankara, Turkey; ^2^Department of Oral and Maxillofacial Surgery, Faculty of Dentistry, Ankara University, 06500 Ankara, Turkey; ^3^Department of Orthodontics, Faculty of Dentistry, Ankara University, 06500 Ankara, Turkey

## Abstract

*Objective*. This case report presents 3-year follow-up of a case of nonsyndromic multiple supernumerary teeth (NSMST) with 11 supernumerary teeth, 2 of which showed subsequent formation. *Case Report*. A 10-year-old girl was referred to the dental clinic with the chief complaint of delayed eruption. Radiographic examination showed 9 retained supernumerary teeth. The treatment plan consisted of extraction of the supernumerary teeth and associated primary teeth in order to allow the permanent teeth to erupt. After 2 years of follow-up, 2 additional supernumerary teeth were observed. *Conclusion*. Regular follow-up for late forming supernumeraries is crucial for NSMST cases.

## 1. Introduction

Supernumerary teeth refer to teeth in excess of the normal full complement of teeth in primary or permanent dentition. The prevalence of supernumerary teeth ranges from 0.1% to 3.6%, and they are twice as common in permanent compared to in primary dentition [1–3].

Supernumerary teeth may occur singly, multiply, unilaterally, or bilaterally in the maxilla, mandible, or both. The anterior maxillary region appears to be the site of predilection [1, 3].

Multiple supernumerary teeth are generally associated with cleidocranial dysplasia, Gardner's syndrome, or cleft lip and palate [2]. Nonsyndromic multiple supernumerary teeth (NSMST) is a rare disorder that describes the presence of five or more supernumerary teeth not associated with another disease such as those mentioned above [2, 3]. The condition has been described in the literature by isolated cases or series of cases [1, 3–5]. The disorder has been reported to occur more frequently in males than in females, with the premolar series the teeth most frequently affected [2, 3, 6]. Various authors have reported cases of NSMST in which supernumerary teeth were seen in the premolar region of the mandible and the anterior-superior and molar region of the maxilla [1–5].

In general, supernumerary teeth begin to develop before the teeth of the dental series to which they are related; however, some authors have described the subsequent formation of supernumerary teeth [3, 7–10].

This case report presents the treatment and 3-year follow-up of NSMST in a 10-year-old girl with 11 supernumerary teeth, 2 of which showed subsequent formation.

## 2. Case Report

A 10-year-old Caucasian girl was referred to the paediatric dentistry clinic with the chief complaint of delayed eruption of the permanent maxillary anterior teeth. There was no significant medical history and no family history of dental anomalies. The patient was a healthy child with no mental retardation, normal facial appearance, and no skeletal or other abnormalities suggestive of a systemic syndrome. She was the first child of a nonconsanguineous marriage, and her parents had no hereditary peculiarities.

**Figure 1:**
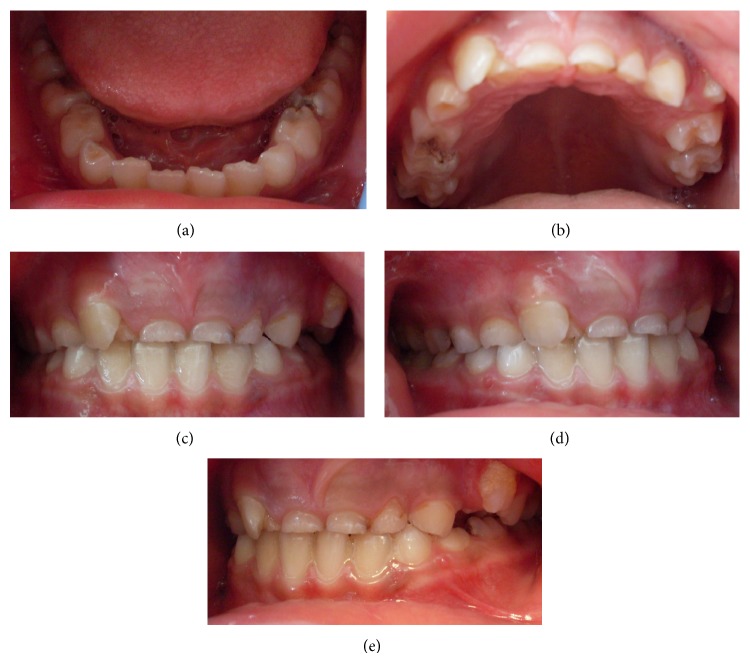
Preoperative intraoral photographs.

Intraoral examination revealed the presence of over-retained primary maxillary anterior teeth. The patient was in the mixed dentition stage and had a Class I molar relationship (Figures 1(a)–1(e)). Oral hygiene was fair, resulting in multiple carious lesions.

**Figure 2:**
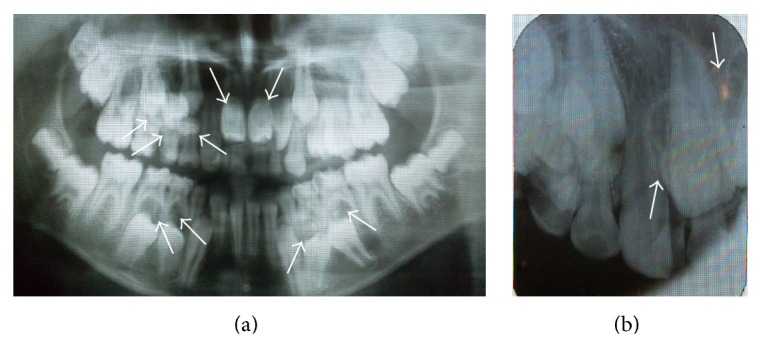
Preoperative radiographs showing multiple supernumerary teeth. (a) The arrows show 9 supernumerary teeth in both jaws. (b) The arrows show 2 supernumerary teeth in the upper anterior region.

Radiographic examination showed 9 retained supernumerary teeth: 2 mesiodens, 4 in the mandibular left and right premolar regions, 2 in the maxillary right premolar region, and 1 in the maxillary right canine region (Figures 2(a)-2(b)). Panoramic radiographs of the patient's parents showed no pathology or supernumerary teeth; however, her 2-year-old brother was not evaluated due to his young age.

**Figure 3:**
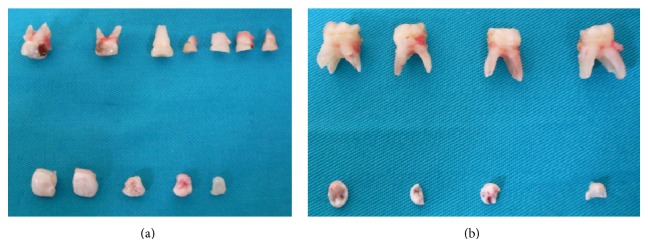
Postsurgical photographs showing extracted teeth. (a) Maxillary extracted primary molars and supernumerary teeth. (b) Mandibular extracted primary molars and supernumerary teeth.

The proposed treatment plan consisted of extraction of the retained supernumerary teeth and associated primary anterior and molar teeth in order to allow the permanent teeth to erupt. The treatment plan was explained to the patient and her family. With their permission, 20 teeth were extracted under general anesthesia (11 primary teeth and 9 impacted supernumerary teeth) (Figures 3(a)-3(b)).

**Figure 4:**
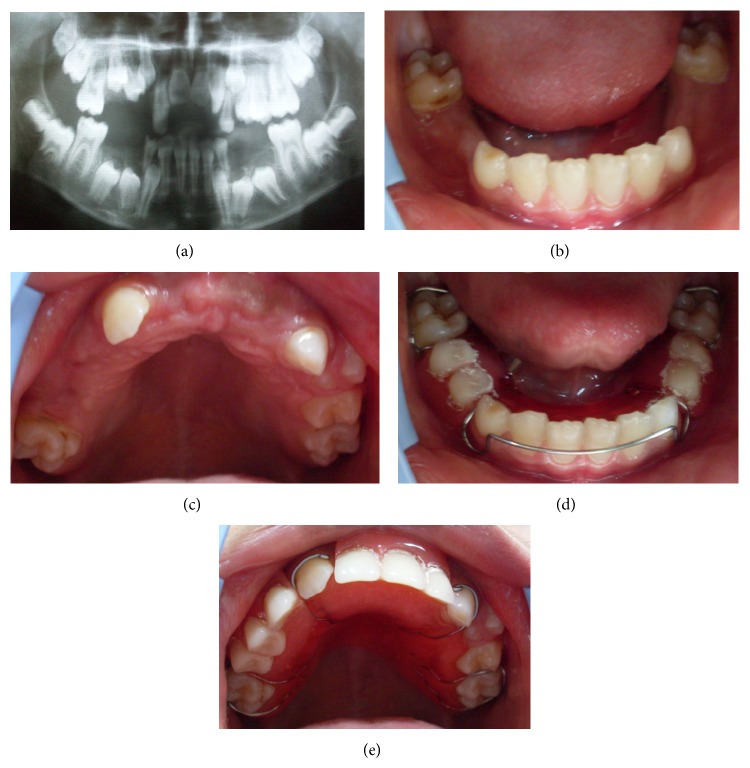
Intraoral and panoramic views 1 month after the extractions. (a) Postoperative panoramic radiograph. (b, c) Postoperative intraoral views of the patient. (d, e) Space maintainers were prepared for maxillary and mandibular regions.

**Figure 5:**
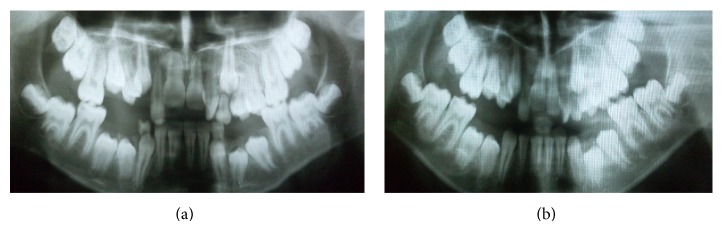
(a, b) Six-month and 12-month follow-up radiographs.

Figures 4(a)–4(e) shows an intraoral view and panoramic radiograph of the patient 1 month after the extractions. The patient was also provided with removable space maintainers and routine clinical and radiographic follow-up was performed at 6, 12, and 24 months in order to detect any possible delayed appearance of new supernumerary teeth.  Figure 5 shows the panoramic radiographs of 6th and 12th months.

**Figure 6:**
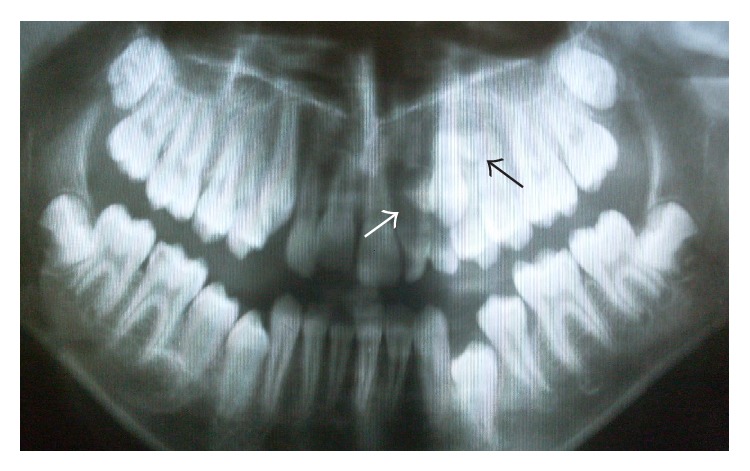
Two-year follow-up radiograph showing 2 additional supernumerary teeth, 1 in the maxillary left premolar region and 1 in the maxillary anterior region.

**Figure 7:**
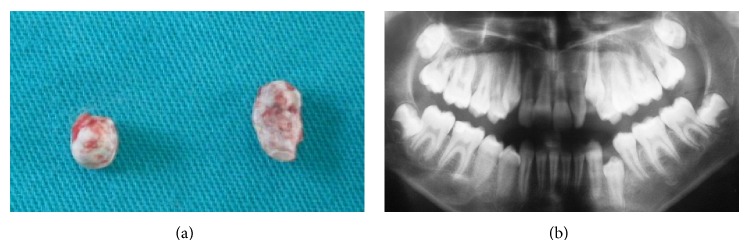
(a, b) Postsurgical photographs of the extracted supernumerary teeth and panoramic view.

After 2 years of follow-up, 2 additional supernumerary teeth were observed, 1 in the maxillary left premolar region and 1 in the maxillary anterior region (Figure 6). In all other regions, eruption of the permanent premolars was normal. Extraction of the new supernumerary teeth was performed (Figures 7(a)-7(b)), and orthodontic treatment was initiated.

**Figure 8:**
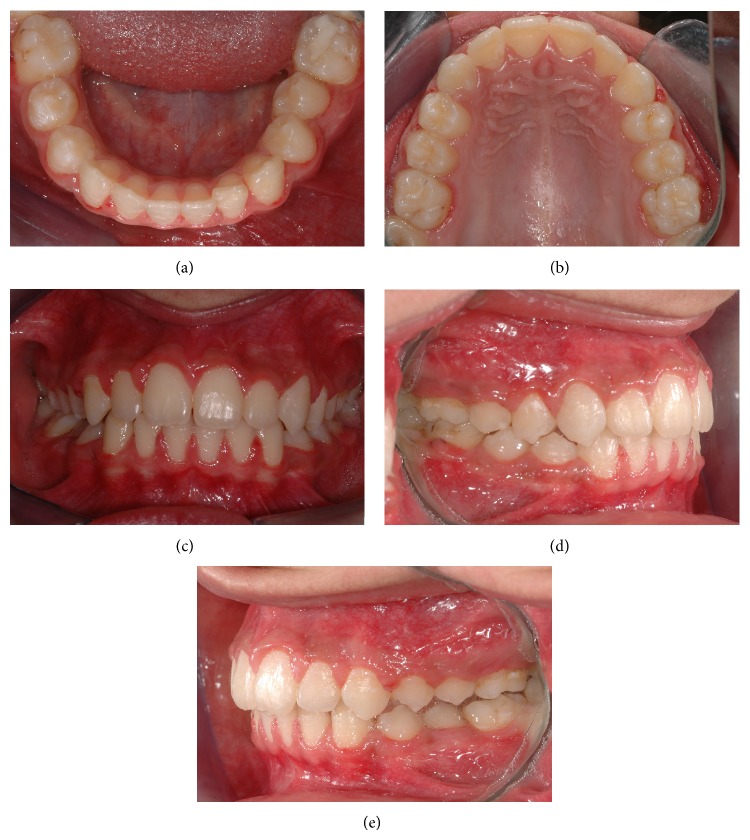
(a–e) Intraoral views of the patient after completion of orthodontic treatment.

**Figure 9:**
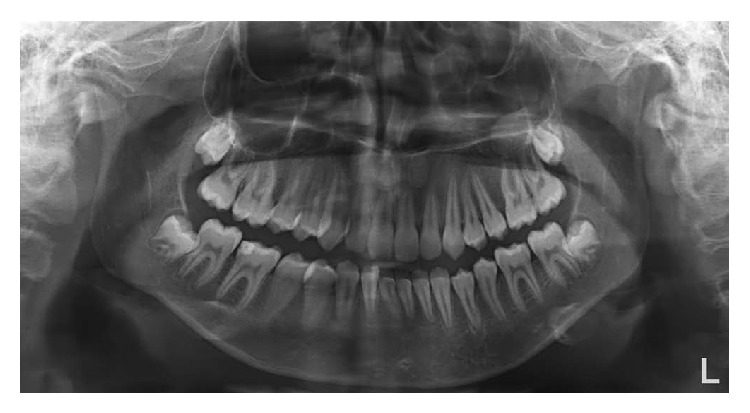
Panoramic view of the patient at the end of 3-year follow-up.

At the end of a 3-year follow-up, orthodontic treatment using fixed mechanics was shown to have achieved good esthetics with an ideal overjet and overbite relation (Figures 8(a)–8(e) and Figure 9). An Essix appliance was constructed for retention.

**Figure 10:**
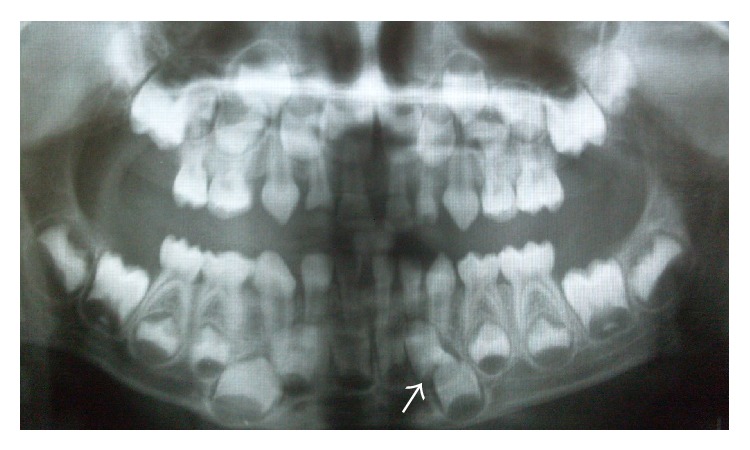
Panoramic radiograph of the patient's brother showing impacted supernumerary tooth.

Panoramic radiograph of the younger brother of the patients' at age 5 showed an impacted supernumerary tooth in the mandibular left canine region (Figure 10). His follow-up visits are also continuing; however the extraction the supernumerary tooth was delayed until the eruption time of premolars.

## 3. Discussion

Although the exact etiology of supernumerary teeth is unknown, several theories have been postulated to try to explain their presence. Of these, the most acceptable appear to be phylogeny (regression to anthropoids, whose dental formula contained more teeth); autonomic recessive inheritance linked to the X chromosome; an abnormal reaction to a local traumatic episode; environmental factors; dichotomy of the tooth germ; and hyperactivity of the dental lamina [1, 2, 6]. Multiple hyperdontia may be associated with a number of syndromes, and multiple supernumerary teeth not associated with syndromes are rare [2, 6]. The diagnosis of NSMST is achieved mainly through clinical and radiographic examination showing the presence of five or more supernumerary teeth, regardless of their location. The case presented here is that of a healthy girl with no systemic disorders and a total of 11 supernumerary teeth.

The literature points to a familial predisposition to hyperdontia [1, 3, 4]. In this case, the patient's parents did not have any supernumerary teeth, and there was no familial history of the disorder; however, her younger brother had 1 supernumerary tooth. This was probably due to the low penetration of autosomal dominant transmission, which implies that some generations are not affected by the disorder.

Yagüe-García et al. reported the majority (76.7%) of supernumerary teeth in NSMST to be located in the upper jaw, with the mesiodens the most frequent supernumerary tooth, representing 35.29% of all supernumerary teeth in NSMST [3]. In the case presented here, 7 out of 11 supernumerary teeth were in the maxilla, and 2 of these were mesiodens.

Late formation of supernumerary teeth has been reported particularly in the premolar region [3, 7–9]. In our case, after 2-year follow up, the development of 2 additional supernumerary teeth was observed; however, one of them was in the anterior region in contrast to other cases. For this reason, conducting periodic radiological examinations to rule out the formation of new supernumerary teeth is advisable in suspected cases of NSMST [3].

Supernumerary teeth may erupt normally, stay impacted, appear inverted, or assume an ectopic position or an abnormal path of eruption. The development of supernumeraries may precipitate a variety of complications, including crowding, delayed eruption or impaction, diastema development, cystic lesions, occlusal problems, and resorption of adjacent teeth. Therefore, early diagnosis, proper evaluation, and appropriate treatment are essential [3, 5, 10]. There is no single ideal treatment option for supernumeraries; rather, treatment may vary from simple extraction to extraction followed by orthodontic treatment [2, 6]. Surgical removal of impacted teeth involves the risk of damage to adjacent structures; therefore, surgical risks and benefits of removal must both be taken into consideration in deciding on treatment [2, 5, 6]. In this case, the supernumerary teeth were extracted because they would impede the eruption of permanent premolars and incisors.

## 4. Conclusion

Regular follow-up for late forming supernumeraries is crucial for NSMST cases. Radiographic monitoring of the siblings is also important in such cases.
